# Understanding the role of *"*sunshine vitamin D*"* in Parkinson’s disease: A review

**DOI:** 10.3389/fphar.2022.993033

**Published:** 2022-12-19

**Authors:** Tapan Behl, Arpita Arora, Rajeev K. Singla, Aayush Sehgal, Hafiz A. Makeen, Mohammed Albratty, Abdulkarim M. Meraya, Asim Najmi, Simona Gabriela Bungau

**Affiliations:** ^1^ School of Health Science and Technology, University of Petroleum and Energy Studies, Bidholi, Uttarakhand, India; ^2^ Chitkara College of Pharmacy, Chitkara University, Rajpura, Punjab, India; ^3^ Institutes for Systems Genetics, Frontiers Science Center for Disease-Related Molecular Network, West China Hospital, Sichuan University, Chengdu, Sichuan, China; ^4^ School of Pharmaceutical Sciences, Lovely Professional University, Phagwara, Punjab, India; ^5^ GHG Khalsa College of Pharmacy, Gurusar Sadhar, Ludhiana, Punjab, India; ^6^ Pharmacy Practice Research Unit, Clinical Pharmacy Department, College of Pharmacy, Jazan University, Jazan, Saudi Arabia; ^7^ Department of Pharmaceutical Chemistry and Pharmacognosy, College of Pharmacy, Jazan University, Jazan, Saudi Arabia; ^8^ Department of Pharmacy, Faculty of Medicine and Pharmacy, University of Oradea, Oradea, Romania; ^9^ Doctoral School of Biomedical Sciences, University of Oradea, Oradea, Romania

**Keywords:** vitamin D, Parkinson’s disease, neuroprotection, supplement, neurological disorder

## Abstract

Next to Alzheimer’s disease, Parkinson’s disease constitutes the second most widespread neurological disorder, primarily affecting the older population. Its symptoms are noticeable with advancing age including tremors, postural imbalance, and slow movements, and over time, these symptoms get aggravated, progressing to osteoporosis, osteopenia, and risk of fractures. These symptoms correlate to low bone density and hence weakened bones; thus, vitamin D proves to be an intricate component of the pathogenesis of the disease. Moreover, lower serum concentrations of vitamin D have been found in diseased subjects. Supplementation with vitamin D can retard the aggravation of non-motor as well as motor symptoms of Parkinson’s disease that include cognitive improvement along with the decline in risk of fractures. Also, vitamin D is extremely crucial for brain functioning, targeting dopaminergic neurons, and almost the entire functioning of the brain is affected. However, further exploration is required to determine the toxic dose of vitamin D in Parkinson’s subjects. This “sunshine vitamin” surely can be a ray of sunshine for neurologically diseased subjects.

## Introduction

The most widespread neurodegenerative disorder other than Alzheimer’s disease is “shaking palsy” or Parkinson’s disease (PD), affecting about one/two people per thousand population. The incidence of developing the disorder increases as age increases, particularly affecting those above the age of 60 years (Tysnes and Storstein, 2017). The recognition of the disorder by the term “shaking palsy” is absolutely precise owing to its characteristic features that include tremors, slow movements, rigidity, and an imbalanced posture. Various sources of evidence suggest that PD patients have an abnormal vitamin D endocrine system, comprising a decline in the vitamin D level and less bone density, along with elevated bone-turnover indicators—urinary N-terminal telo-peptide and alkaline phosphatase of the bone (Abou-Raya et al., 2009). These parameters combined with postural imbalances contribute to the occurrence of fractures in diseased subjects, particularly hip fractures among geriatric females (Sato et al., 2001).

Vitamin D, a lipid-soluble steroid, the site of synthesis being the skin, is generated primarily *via* exposure to sunlight and also *via* dietary components (Cesari et al., 2011). Activation of the vitamin occurs *via* hydroxylation which occurs twice inside our body. The foremost hydroxylation site is the liver, converting vitamin D to 25-hydroxy vitamin D, and the other one is the kidneys, where 25-hydroxy vitamin D is transformed into calcitriol/1,25-dihydroxy vitamin D, which is the active form of vitamin D, and the effects are seen when it communicates with the receptor for vitamin D (Cesari et al., 2011). This “sunshine vitamin” proves to be extremely essential for the therapy of rheumatoid arthritis, asthma (Rappaport et al., 1934; Scheuring et al., 2007), cancer, and neurodegenerative disorders, such as PD (Bouillon, 2018). There have been numerous studies correlating vitamin D levels and PD; for instance, the patients with PD usually suffer from osteoporosis (more commonly observed in women than in men) (Invernizzi et al., 2009), leading to elevated chances of hip fractures owing to lower bone density along with low calcium (Sato et al., 2001). Also, vitamin D levels are notably lower in PD subjects than those in healthy subjects (Peterson et al., 2013a; Rimmelzwaan et al., 2016), and the level of 25-hydroxy vitamin D exhibits a gradual decline as the motor signs of PD augment (Van den Bos et al., 2013).

There occur a plethora of mechanisms that correlate neurodegenerative disorders with the “sunshine vitamin.” The vitamin plays a vital role in neuronal protection *via* nerve growth regulation, as well as *via* neurotrophic factors (Garcion et al., 2002). In fact, 1,25-dihydroxy vitamin D3 can synthesize neurotrophic factors (derived from cell lines of glial cells) as well as neurotrophin 3 (Wion et al., 1991; Brown et al., 2003), resulting in neuroprotection as observed in rats (Riaz et al., 1999; Wang et al., 2000). The toxic effects of reactive oxygen species (ROS) are also affected by vitamin D. Our immune system produces inducible nitric oxide synthase inside nerve cells along with other cells of the central nervous system (Garcion et al., 1998). Higher concentrations of nitric oxide exert a damaging effect on the nerve cells. The interesting component here is that the presence of vitamin D has an inhibitory effect on inducible nitric oxide synthase (Garcion et al., 1998). Also, in the nerve cells of the hippocampus, vitamin D downregulates the calcium ion channels (voltage sensitive) exhibiting protective effects on the neurons (Li et al., 2011). Yet another crucial factor is the reduction of oxidative stress, leading to nerve cell protection by the vitamin and causing a decline in cell death (Ibi et al., 2001). The intricate relationship between vitamin D and PD was initially identified in 1997 by Sato et al. (1997). The substantia nigra of the brain contains the receptor for vitamin D and the prominent vitamin D-activating enzyme, 1α-hydroxylase, that accounts for the fact that the deficiency of the vitamin progresses to the dysfunctional substantia nigra, the primary affected part in PD (Shen et al., 2004).

Here, we review the metabolism of vitamin D, declining concentrations of vitamin D in PD subjects, important functions of the vitamin in the brain, and its association to clinical manifestations of PD coupled with the fundamentals for administering vitamin D3 to PD subjects, utilizing the method of reviewing the referenced reviews and research papers, searching the material through the usage of appropriate keywords, and stating the facts accordingly. Moreover, complete utilization of vitamin D in PD therapy could be carried out by overcoming certain limitations mentioned in the conclusion for which adequate data stand unavailable (Higgins et al., 2019).

## Vitamin D: Biotransformation and receptor interaction

The fundamental source of Vitamin D, also called “sunshine vitamin,” is sunlight. Apart from this, the vitamin can be obtained *via* diet after which the liver enzyme, 25-hydroxylase, transforms it into 25-hydroxy vitamin D, and this constitutes the vitamin in its circulatory form. Moreover, 25-hydroxy vitamin D serves as a biological marker to detect serum levels of the vitamin in people with PD, but this circulatory form is not the active form and thus necessitates its transformation *via* 25-hydroxy vitamin D-one-alpha hydroxylase enzyme to 1,25-dihydroxy vitamin D3 (process occurs inside the kidney), which is the activated form of the vitamin. This active form, when present at extremely elevated levels, can be further metabolized to calcitroic acid *via* the metabolic enzyme 25-hydroxy vitamin D 24 hydroxylase (Lv et al., 2020).

**FIGURE 1:**
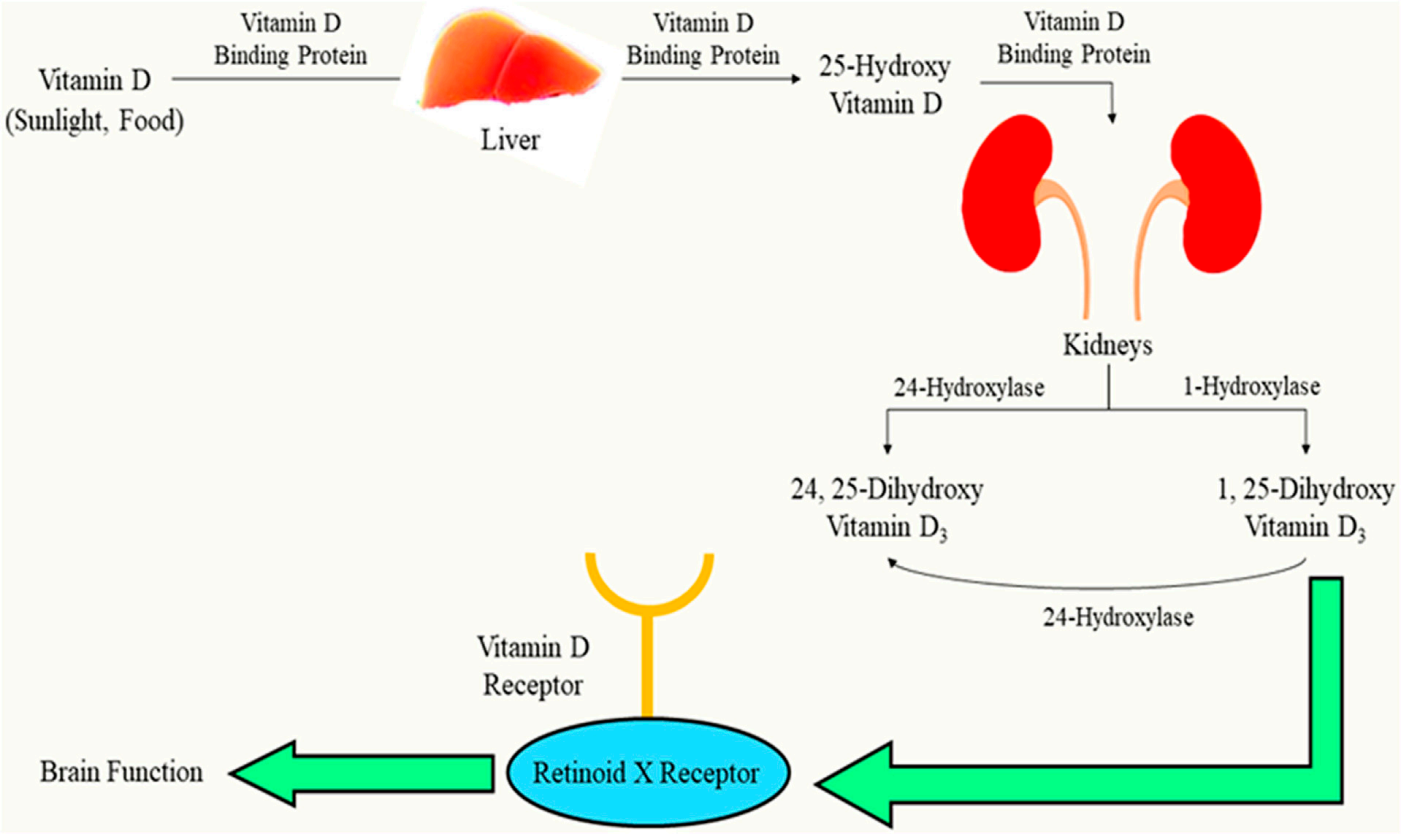
Biotransformation of vitamin D and interaction with its receptor facilitating brain function.

1,25-Dihydroxy vitamin D3 exhibits its actions through an interaction with the receptor for the vitamin, majorly confined to the nucleus since it is a nuclear receptor (DeLuca, 2004). When a ligand is attached to the receptor, the receptor exhibits an interaction with the retinoid X-receptor, resulting in the formation of a hetero-dimer. The hetero-dimer and vitamin D response elements interact, thus promoting latter’s expression (Holick, 2007). This biotransformation has been indicated in Figure 1.

## Vitamin D and PD in parallel: Low vitamin D levels in PD subjects

Vitamin D deficiency has become extremely common throughout the world, especially in the aged population (Dawson-Hughes et al., 2010), and recent research claims that the deficiency of the vitamin is found to be closely associated with neurodegenerative disorders such as PD (Ding et al., 2013). Probably, deficiency of vitamin D does not occur as a result of neurodegenerative diseases, and this is evident from the fact that in the case of Alzheimer’s disease, vitamin D levels are not significantly low, whereas vitamin D levels are found to be considerably low in PD (Evatt et al., 2008). The concentration of 1,25-dihydroxy vitamin D3 does not decrease in PD subjects, but the concentration of 25-hydroxy vitamin D falls significantly in serum to as low as 20 ng per milliliter (according to prospective cohort studies) (Suzuki et al., 2012). The probable cause for decreased 25-hydroxy vitamin D is that its levels in serum/blood are about a thousand times greater than 1,25-dihydroxy vitamin D3 (Suzuki et al., 2012).

There were several studies to explore the possible causes of the deficit in vitamin concentration in diseased subjects. A study came up with one of the reasons as decreased motility of PD patients and the longer course of the disease that ultimately reduces the probability of getting exposed to sunlight, leading to a decline in the synthesis of the vitamin (Suzuki et al., 2012; Peterson, 2014; Zhou et al., 2019). However, another study came up with the fact that even in PD patients who had adequate sun exposure, vitamin D levels were low owing to a decline in gastrointestinal functioning (Mueller et al., 2021). Such lowering of vitamin D levels due to a decline in gastrointestinal function was overruled by another study because 25-hydroxy vitamin D was higher in plasma and hypovitaminosis is much more common in patients with “early PD” (Evatt et al., 2011).

## Vitamin D and brain functioning: An intricate association

Enzymes involved in the biological synthesis of the sunshine vitamin (25-hydroxylase, etc.) as well as the receptor for vitamin D are presented in the embryonic brain and adult brain (Eyles et al., 2005; de Abreu et al., 2009). The receptor for the vitamin is present in abundance in the amygdala, thalamus, cortex, and hippocampus (Stumpf et al., 1982) with 1α-hydroxylase being present in abundance in the substantia nigra. Vitamin D facilitates the regulation of multiplication, differentiation, and the viability of the cells *via* which it is synthesized, i.e., microglia and nerve cells (de Abreu et al., 2009). This vitamin also plays a crucial role in providing synaptic plasticity as well as in ameliorating brain function (Almeras et al., 2007), and this synaptic plasticity is provided by the vitamin owing to its role in the regulation of protein expression including connexin 43, growth-associated protein 43, and drebrin; transportation of kinesin, creatine kinase B, and dynactin; and maintaining the cytoskeletal structure comprising neuro-filament, tubulin, and microtubule-associated protein 2 (Almeras et al., 2007; de Abreu et al., 2009). A preclinical study showed that lower vitamin D levels at the maternal stage cause alterations in the brain such as reduced cortical thickness and anatomically increased lateral ventricle in the newborn (Eyles et al., 2003; Grecksch et al., 2009).

Vitamin D seems to be engaged in neuroprotection since the lowered level of 25-hydroxy vitamin D causes dopaminergic nerve cell death, leading to PD, and also the functions mentioned previously exhibit its crucial role in brain functioning (Suzuki et al., 2012), and there are numerous mechanisms in support of this fact. Various mechanisms state that the production of parvalbumin (calcium ion-binding protein), as well as the release of neurotrophin, is stimulated *via* 1,25-dihydroxy vitamin D3; gamma-glutamyl transpeptidase (necessary for the regulation of calcium ion balance and anti-oxidant effect) is upregulated, while the L-type voltage-sensitive calcium ion channel is downregulated (necessary for neurotransmission); and calcitriol does not permit the formation of inducible nitric oxide synthase, tumor necrosis factor *α*, and macrophage colony-stimulating factor (Brewer et al., 2001; de Sire et al., 2022). Moreover, when calcitriol is lower in the body, there occurs a rise in inflammation which is indicated by higher levels of C-reactive protein (Alfieri et al., 2017). Not only this, there are certain growth factors that the vitamin synthesizes such as ciliary neurotrophic factor and brain-derived neurotrophic factor, among others, that prevent the brain from aging and degenerating (de Sire et al., 2022). The vitamin has a critical function of maintaining calcium ion concentration in the neurons as well as in the glial cells. If this calcium ion concentration is not maintained, it will probably cause excitotoxicity in the cytoplasm due to a spike in the calcium ion concentration; hence, the injury due to excitotoxicity is avoided (Brewer et al., 2001). By reducing the production of inducible nitric oxide synthase and nuclear factor kappa-light-chain-enhancer of activated B cells (NF-kB), as well as incrementing the activity of gamma-glutamyl transpeptidase, the oxidative stress is significantly reduced (van Etten and Mathieu, 2005). Nonetheless, significantly lower vitamin D levels can actually progress to impaired functioning of the neurons of the sympathetic nervous system due to the involvement of the vitamin in the functioning of the renin–angiotensin–aldosterone system/RAAS (Ometto et al., 2016).

Vitamin D plays a major role in the functioning of the cerebrum to transmit signals in the nervous system in order to facilitate locomotor, emotional, and rewarding behavior along with intelligence. All of this is dopamine level-dependent and under the influence of vitamin D (Trinko et al., 2016). The site of dopamine production in the substantia nigra and into the tegmental area (ventral) further projects into the striatum (dorsal part) and the prefrontal cortex. The dorsal striatum participates in motor activities, whereas the tegmental area (ventral), as well as the prefrontal cortex, participates in reward behavior. When the receptor for vitamin D is overexpressed in the striatum (as seen in preclinical studies), an elevation in motivation as well as reward behaviors occurs (Burne et al., 2006; Zhang et al., 2018). Vitamin D affects locomotion; thus, the mice that do not have the receptor for vitamin D showing dysfunction in motor performance (vinh quôc Luong and Thi Hoàng Nguyên, 2012). The vitamin also plays a role in the regulation of emotions and mood owing to its presence in the hippocampus, cortex, and amygdala (i.e., limbic system) (Bertone-Johnson, 2009).

**FIGURE 2:**
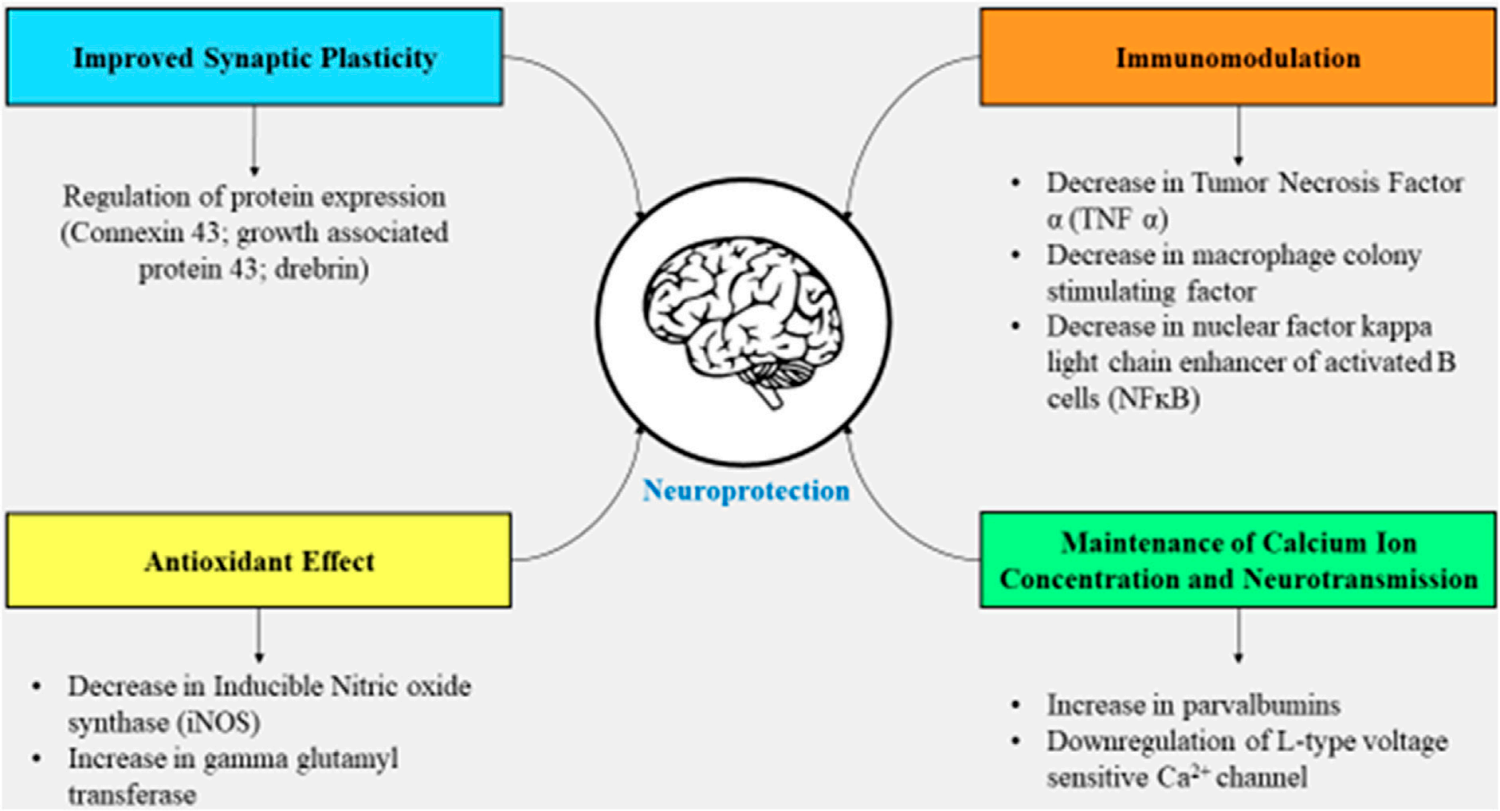
Effects of vitamin D on the brain facilitating synaptic plasticity, immunomodulation, neurotransmission, and a decline in oxidative stress.

Vitamin D and its receptor facilitate the proper functioning of the neurons as mentioned previously, and to further strengthen the neuronal circuit, vitamin D also influences the generation of serotonin (Patrick and Ames, 2015; Penckofer et al., 2017). The intricate association of vitamin D with brain functioning is summarized in Figure 2.

## Vitamin D: A neuroprotective agent

The re-growth of neurons, as well as the protection of dopaminergic nerve endings, is facilitated *via* glial cell-derived neurotrophic factors (GDNFs), and hence, the GDNFs have the potential for neuro-restoration as a part of treatment for PD (Weissmiller and Wu, 2012; Lv et al., 2020). The receptor, known as GDNF family receptor alpha 1 (GFRA1), is the binding site for GDNFs, further associating with receptor-tyrosine protein kinase Ret/C-Ret (a proto-oncogene receptor). The complexation favors signaling within the dopaminergic nerve cells (Pertile et al., 2018). Although GDNF has the potential for neuro-restoration, its inability to cross the blood–brain barrier (BBB) necessitates the need to be injected into the central nervous system, leading to numerous adverse effects (Lv et al., 2020). Thus, to overcome this limitation, administering 1,25-dihydroxy vitamin D3 is the solution, and as being lipid-soluble, it can penetrate the BBB (DeLuca et al., 2013), indicating the vital role of this hormone in the therapy of PD. 1, 25-Dihydroxy vitamin D3 further binds to the receptor for vitamin D upregulating the transcription of genes stimulated *via* GDNF, as well as C-Ret, both of which can decrease the generation of GDNF family receptor alpha-1 (GFRA1) (Pertile et al., 2018). The functional specificity, as well as maturity of dopaminergic nerve cells, in mice is affected (Cui et al., 2010) due to the decreased levels of calcitriol that lower the relative abundance of nuclear receptor-related 1 protein (Nurr1), p57kip2, and GDNF (Eyles et al., 2003; McGrath et al., 2004). C-Ret that activates tyrosine kinase enzyme necessary for mechanisms, such as phosphor-inositol 3 kinase (PI3K), phospholipase 3 pathway, and mitogen-activated protein kinase, is also expressed when nuclear receptor-related 1 protein exhibits its presence (Sariola and Saarma, 2003; García-Martínez et al., 2006). The activation of these mechanisms is crucial for the dopaminergic neurons to survive and acquire functional specifications.

Apart from augmenting the presence of the *GDNF* gene and activating the mechanisms, neuroprotection is also offered by 1, 25-dihydroxy vitamin D3 by exerting anti-oxidant effects since GDNF has the capacity to scavenge free radicals *via* augmentation of catalase and glutathione peroxidase enzymes in the striatum nigra (Chao and Lee, 1999). The active form of the vitamin exhibits its anti-oxidant potential *via* genomic and non-genomic mechanisms. When inflammation strikes in, there occurs *in situ* generation of calcitriol by microglia enhancing the activity of gamma-glutamyl transferase. The gamma-glutamyl transferase enzyme further promotes glutathione in the cells that exert an anti-oxidant effect (scavenges hydrogen peroxide) (Garcion et al., 1999). The nuclear factor-erythroid 2-related factor 2 (Nrf2) attaches to anti-oxidant response elements (AREs) inside the nucleus that augments the presence of anti-oxidant genes along with *fos* proto-oncogene (Fos) and JUN (Berridge, 2015). Another crucial benefit of administering 1, 25-dihydroxy vitamin D3 is that cell membranes are protected from damage due to oxidative stress due to the inhibition of peroxidation of lipids (Wiseman, 1993).

**FIGURE 3:**
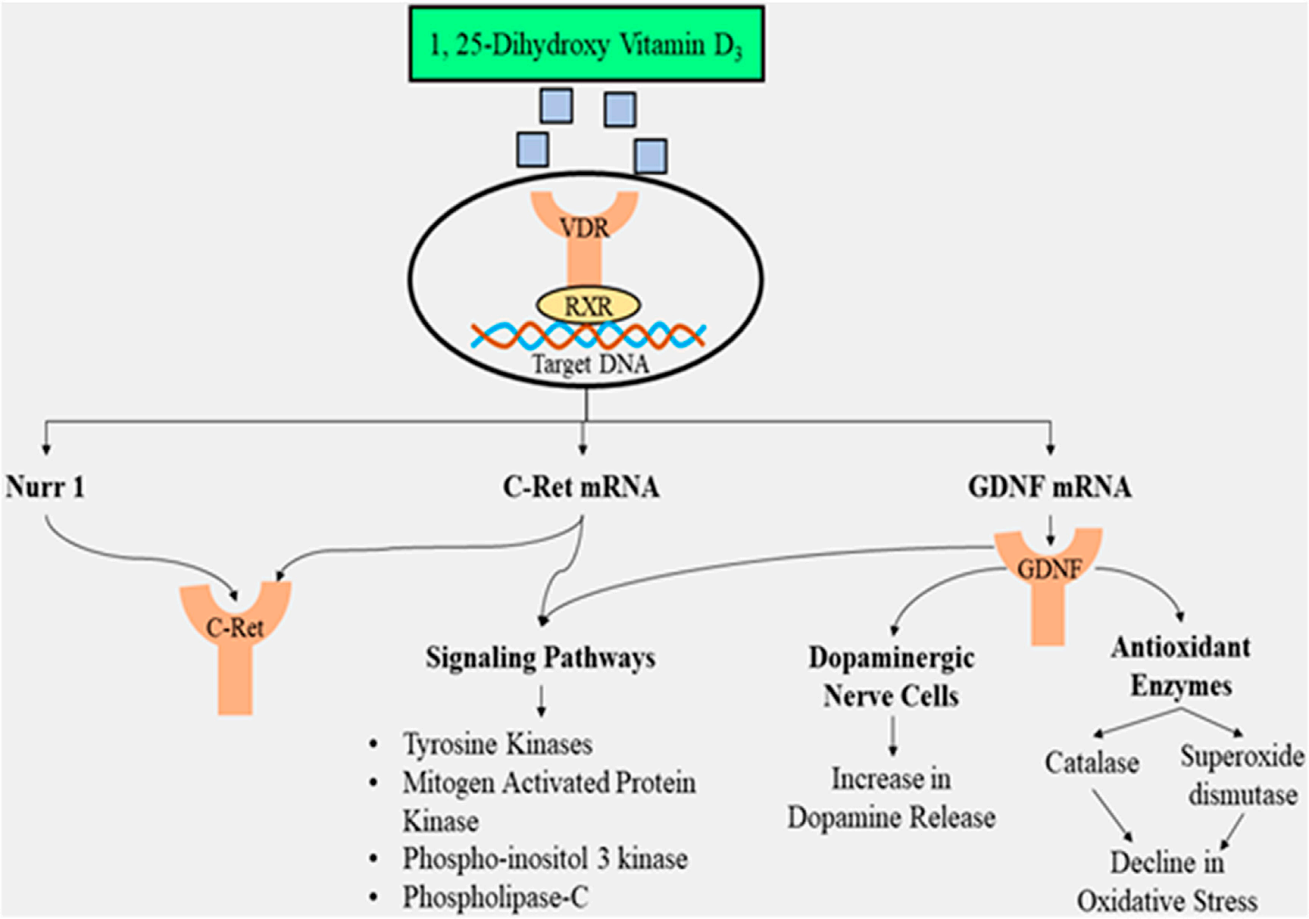
Illustration of 1,25-dihydroxy vitamin D3 as a neuroprotective agent preventing dopaminergic neuronal loss and suppressing oxidative stress along with triggering multiple signaling pathways.

The active form of the vitamin also possesses anti-inflammatory properties by decreasing the inflammatory activities and augmenting the anti-inflammatory activities (Calvello et al., 2017). According to a cell culture study, the increased levels of calcium ions within the cell induce clumping of alpha-synuclein (Rcom-H’cheo-Gauthier et al., 2017). In order to maintain decreased calcium ion levels in the cytosol, the presence of L-type calcium ion channels has to be reduced, augmenting the levels of calcium-ATPase, Bcl2 (apoptosis regulator), sodium–calcium exchanger 1(NCX1), and calbindin-D28k (buffering protein) (Berridge, 2015; Phillipson, 2017). A factor that contributes to the increased oxidative stress is increased levels of metal ions such as zinc and manganese, and the level of these ions is maintained by 1,25-dihydroxy vitamin D3 by increasing their transportation *via* activation of *SLC30A10* that augments the presence of the transporter ZnT10 (zinc transporter) (da Silva et al., 2016). This will reduce oxidative stress and damage to mitochondria as well as cell membranes. The illustration of 1,25-dihydroxy vitamin D3 as a neuroprotective agent is depicted in Figure 3.

The impact of calcitriol on various genes is summarized in Table 1.

**TABLE 1:** Influence of 1,25-dihydroxy vitamin D3 on the expression (upregulation/downregulation) of genes.

Gene	Functions in the brain	Impact on the expression of gene	Reference
*GDNF*	Neuroprotective and anti-oxidant	Upregulation	Pertile et al. (2018)
*C-Ret*	Anti-oxidant and neuroprotective	Upregulation	Pertile et al. (2018)
*p57kip2*	Functional specification and maturation of dopaminergic nerve cells	Upregulation	Cui et al. (2010); Luong and Nguyen (2012)
*Nurr1*	Functional specification and maturation of dopaminergic nerve cells	Upregulation	Cui et al. (2010); Luong and Nguyen (2012)
*SLC30A10*	Maintains levels of zinc, magnesium, calcium, and iron	Upregulation	da Silva et al. (2016)

## Clinical findings in PD patients: Vitamin D therapy in PD subjects as a valuable approach

Clinical manifestations that occur in PD patients directly indicate the need for vitamin D supplementation, and this section throws light on some of these.

People who suffer from PD have increased chances of developing osteopenia along with osteoporosis (Invernizzi et al., 2009). Moreover, patients with PD can develop secondary osteoporosis (Bezza et al., 2008). According to one research conducted in Korea, out of the total 35,663 patients participated in the study, about 6,542 patients developed osteoporosis and developed fractures 6 months post-development of PD (Park et al., 2019). Patients with PD have more chances of loss of bone density, falls, and hip fractures (Lyell et al., 2015); thus, timely therapy for osteoporosis should begin, especially for elderly women (Park et al., 2019). Due to these reasons, another study came up with the fact that the administration of 700–1,000 IU (international units) of vitamin D can possibly lower the chances of falling by 19 percent without the need for calcium intake (Bischoff-Ferrari et al., 2009). Further research has established a negative correlation between the intensity of disease developed (as measured by the Unified Parkinson’s Disease Rating Scale) and the concentration of 25-hydroxy vitamin D in plasma (Suzuki et al., 2012; Chitsaz et al., 2013; Peterson, 2014; Zhou et al., 2019). Hence, treatment *via* vitamin D3 can retard aggravating PD symptoms (Peterson et al., 2013b). Also, a relationship exists between lower vitamin D levels and a decrease in intelligence and memory, progressing to the risk of dementia (Feart et al., 2017). This comes with the fact that was proved by yet another study that elevated levels of vitamin D improve memory and behavior (Peterson et al., 2013a). Vitamin D has an impact on memory because it affects amyloid beta protein (Hooshmand et al., 2014) that accumulates not only in Alzheimer’s but also in PD, progressing to decreased memory (Jellinger et al., 2008). According to a double-blind trial, therapy with vitamin D lowers the concentration of amyloid precursor protein as well as amyloid precursor protein mRNA (Jia et al., 2019). The lack of vitamin D alters the plasticity of synapses, thus causing decreased memory (Mayne and Burne, 2019).

Thus, in a nutshell, vitamin D3 is definitely a critical component of the pathophysiology encompassing PD and hence needs to be incorporated into PD therapy.

The major studies on the administration of the vitamin in people who suffer from PD are listed in Table 2.

**TABLE 2:** Studies indicating the administration of vitamin D3 as a supplement.

Region	Study design	Participants in the treatment group	Participants in the control group	Dose administered	Follow-up time	Reference
Japan	Randomized controlled trial	56	58	1,200 international units per day	1 year	Lv et al. (2020)
The United States of America	Randomized controlled trial	27	24	10,000 international units per day	4 months	Lv et al. (2020)

Vitamin D significantly improves posture in patients with PD when administered daily, especially in the not-so-old PD patients (Hiller et al., 2018). There have been other research studies that mention a positive impact of administering 25-hydroxy vitamin D on geriatric PD patients (Muir and Montero-Odasso, 2011). However, whether the administration of vitamin D supplement has a specificity for delaying motor symptoms in PD patients or acts non-specifically to improve posture and balance is still a debatable issue (Torres-Oviedo and Ting, 2007; Asaka et al., 2011). An important thing to mention is that vitamin D as a supplement acts depending on the age of PD patients (Hiller et al., 2018). Although several drugs are available for PD and more drugs can possibly be discovered by the technique of “elicitation *via* abiotic stress” (Hassan et al., 2021), vitamin D can be used as a supplement along with these drugs to prevent aggravation of PD symptoms. Moreover, vitamin D3 can also be obtained from herbs as herbal drugs, which display numerous pharmacological activities (Hassan et al., 2022).

Table 3 represents the literature summary table depicting relationship between PD and vitamin D.

**TABLE 3:** Literature summary table: highlighting key points that indicate the co-relation between PD and vitamin D.

Author	Title	Source	Finding
DeLuca et al. (2013)	“Review: The role of vitamin D in nervous system health and disease”	Neuropathology and Applied Neurobiology	Vitamin D is vital for neuroprotection, neuroplasticity, and neurotransmission, influencing cellular processes and expression of genes *via* vitamin D response elements
Bezza et al. (2008)	“Prevalence and risk factors of osteoporosis in patients with Parkinson’s disease”	Rheumatology International	Parkinson’s disease patients have a greater probability of developing osteoporosis/osteopenia, a risk that increases with the severity of the disease
Van den Bos et al. (2013)	“Bone mineral density and vitamin D status in Parkinson’s disease patients”	Journal of Neurology	Patients of Parkinson’s disease have greater chances of bone loss than other people along with declined vitamin D levels. This fact necessitates the importance of screening for vitamin D status and supplementation in the early stages of the disease
Brewer et al. (2001)	“Vitamin D hormone confers neuroprotection in parallel with downregulation of L-type calcium channel expression in hippocampal neurons”	Journal of Neuroscience	Chronic therapy with vitamin D3 offers neuroprotection as observed in animal models and targets L-type voltage-sensitive calcium channels in the hippocampus
Hiller et al. (2018)	“A randomized, controlled pilot study of the effects of vitamin D supplementation on balance in Parkinson’s disease: Does age matter?”	PLOS ONE	Vitamin D therapy possesses the ability to improve postural balance, but high dose supplementation needs further evaluation on balance improvement

## Conclusion and future perspectives

Vitamin D levels are low in patients suffering from PD, and boosting vitamin D levels indicates the possibility of improving mood, cognition, and behavior in PD patients along with preventing the aggravation of PD symptoms. The review convinces us to accept the fact that improving vitamin D levels reduces the incidence of fractures, especially hip fractures, and recovers bone density as well. At the same time, certain limitations that need to be taken into consideration for further research include the necessity to establish the effectiveness of vitamin D3 as a supplement in PD, and determining the correlation between vitamin D3 and PD will be crucial because vitamin D can act as a biomarker for PD. Also, deficiency of the vitamin has become frequent across the globe, and supplements of vitamin D are easily accessible and affordable. Thus, vitamin D can act as a potential candidate that can be used as a supplement in PD; however, another limitation to be taken into account is its toxicity profile, especially in PD subjects, and at exactly what stage of the disease is the supplementation useful.
